# Tolerability and Clinical Outcomes With Anesthesia Dose Reduction During Electroconvulsive Therapy

**DOI:** 10.1001/jamanetworkopen.2024.62054

**Published:** 2025-02-27

**Authors:** Caroline W. Espinola, Tyler S. Kaster, Jake Prillo, Lorina Zapf, Hyewon H. Lee, Karen Foley, Martin Ma, Adriaan Van Rensburg, Daniel M. Blumberger

**Affiliations:** 1 Temerty Centre for Therapeutic Brain Intervention, Centre for Addiction and Mental Health, Toronto, Ontario, Canada; 2 Department of Psychiatry, Temerty Faculty of Medicine, University of Toronto, Toronto, Ontario, Canada; 3 Toronto General Hospital, University Health Network, Toronto, Ontario, Canada; 4 Department of Anesthesiology and Pain Medicine, Temerty Faculty of Medicine, University of Toronto, Toronto, Ontario, Canada

## Abstract

**Importance:**

Electroconvulsive therapy (ECT) practice during the COVID-19 pandemic necessitated restriction of bag-valve mask (BVM) ventilation to minimize disease transmission through aerosolization. To reduce BVM use, many sites decreased doses of anesthesia agents, but the associations of such dose reduction with clinical and tolerability outcomes are unknown.

**Objectives:**

To investigate the association of COVID-19 pandemic modifications in ECT, including reduction in anesthetic dosage and BVM use, with the rates of clinical complications and clinical outcomes.

**Design, Setting, and Participants:**

This retrospective cohort study included data from patients receiving an index course of ECT for any clinical indication treated with methohexital and succinylcholine between March 16, 2017, and March 15, 2023, at the Centre for Addiction and Mental Health in Toronto, Ontario, Canada. Data analysis occurred from February to December 2024.

**Exposure:**

Participants were split into 2 cohorts: pre–COVID-19 (treated between March 2017 and March 2020) and COVID-19 (treated between June 2020 and March 2023).

**Main Outcomes and Measures:**

The primary outcomes were BVM use and rates of complications (specifically, aspiration, bradycardia, insufficient initial sedation dosage, postictal agitation [PIA], residual weakness, and prolonged seizures). Secondary outcomes were rates of response and cognitive impairment. Multivariable logistic regression was used to assess associations of COVID-19 pandemic modifications with outcomes.

**Results:**

A total of 616 individuals (median [IQR] age, 45.0 [31.0-59.0] years; 342 female [55.5%]) were included in the analysis, with 362 in the pre-COVID-19 group and 254 in the COVID-19 group. Demographic and clinical characteristics were similar. The COVID-19 group received lower median (IQR) methohexital dosages (0.82 [0.72-0.96] mg/kg vs 0.87 [0.78-1.00] mg/kg; *P* = .007) and succinylcholine (0.33 [0.27-0.39] mg/kg vs 0.53 [0.45-0.59] mg/kg; *P* < .001) than the pre–COVID-19 group. The COVID-19 group had higher odds of insufficient initial sedation dosage (odds ratio, 2.16, 95% CI, 1.09-4.38) and PIA (odds ratio, 2.81; 95% CI, 1.76-4.56), with no difference for other complications. BVM use was reduced from standard use to 13.8% (35 individuals) during COVID-19. Groups were similar in the rates of response (pre–COVID-19, 246 individuals [68.0%]; COVID-19, 181 individuals [71.3%]; *P* = .38) and cognitive impairment (pre–COVID-19, 75 individuals [20.7%]; COVID-19, 49 individuals [19.3%]; *P* = .66).

**Conclusions and Relevance:**

In this retrospective cohort study, reduction of anesthesia agents was associated with reduced BVM use, with similar clinical outcomes and tolerability other than increased rates of insufficient initial sedation dosage and PIA, suggesting that management of PIA with this anesthesia dosing strategy warrants further investigation.

## Key Points

**Question:**

Were modifications in anesthesia practice in electroconvulsive therapy (ECT) during the COVID-19 pandemic associated with differences in clinical and tolerability outcomes?

**Findings:**

In this cohort of 616 patients receiving acute ECT, COVID-19–related reduced doses of anesthetic agents were associated with reduced bag-valve mask ventilation, with similar effectiveness and tolerability other than increased rates of postictal agitation and insufficient initial sedation dosage.

**Meaning:**

These findings suggest that reduced anesthesia medications are safe for future pandemics provided postictal agitation is addressed and sedation is optimized.

## Teaser

This cohort study assesses associations of modifications in anesthesia practice during the COVID-19 pandemic with differences in clinical and tolerability outcomes among individuals receiving electroconvulsive therapy in Canada.

## Introduction

Since its discovery more than 80 years ago, electroconvulsive therapy (ECT) remains an essential and often life-saving treatment for severe psychiatric symptoms such as acute suicidality and catatonia.^1,2,3,4,5^ Anesthesia practice in ECT involves administration of a general anesthetic, a muscle relaxant (typically succinylcholine), and bag-valve mask (BVM) ventilation to promote hyperoxygenation and to maintain oxygen saturation during the apneic period following muscle relaxant use. ECT is considered an aerosol-generating medical procedure due to the standard use of BVM.^6,7,8^ At the onset of the COVID-19 pandemic, ECT services had to mitigate the risk of virus transmission,^9^ and consequently, provision of ECT was restricted in most services (53%-64%) and suspended in 24% to 27% of centers.^1,2,10,11^

Various modifications were implemented to minimize virus transmission and maintain ECT services during the COVID-19 pandemic,^2,12^ including reduction of treatments, because ECT was often restricted to urgent cases.^12,13^ In addition to standard mandatory measures,^1,2,3,6,7,12,14,15^ anesthesia practice in ECT was also modified. To minimize the potential risk of virus aerosolization, preoxygenation with a facemask was widely adopted,^6,7,12,14,16^ with BVM use restricted to desaturation,^2,6,9,12^ often with a high-efficiency particulate air filter in both scenarios. Standard dosage guidelines recommend 0.5 to 1.5mg/kg for methohexital,^17^ 0.15 to 0.3mg/kg for etomidate,^17^ 0.75 to 2.5 mg/kg for propofol,^17^ and 0.5 to 1.0 mg/kg for succinylcholine,^17,18^ although there is a wide variability in individual response to the latter.^19,20^

An expert consensus on COVID-19 response recommended succinylcholine dosage at the lower end to allow for a faster recovery of spontaneous breathing.^12^ To reduce the apneic period and subsequent BVM use,^8,12^ some services decreased succinylcholine dosage^12,16^ whereas other centers did not.^7^ Our ECT service reduced the dosage of succinylcholine and methohexital. However, the association of these modifications to anesthesia dosage with the tolerability and effectiveness of ECT during COVID-19 is unknown. As such, we used a retrospective cohort design to examine whether COVID-19–related modified anesthesia practice was associated with different rates of BVM use and clinical complications during an acute course of ECT (a course of treatment aimed at targeting severe symptoms of specific disorders such as depression), consisting of 8 to 12 ECT sessions delivered twice or thrice weekly.^21^ Our secondary aim was to assess the association of such modifications with clinical and cognitive outcomes.

## Methods

### Study Design and Data Source

This cohort study was approved by the Centre for Addiction and Mental Health research ethics board; reporting of the study followed the Strengthening the Reporting of Observational Studies in Epidemiology (STROBE) reporting guideline. We created a cohort of individuals with acute ECT treatments delivered between March 16, 2017, and March 15, 2023, at the Centre for Addiction and Mental Health in Toronto, Ontario, Canada. Inpatients and outpatients receiving an acute course of ECT for any clinical indication were included; written informed consent was not required by the research ethics board because this was a retrospective study of clinical care. Two independent cohorts, (1) pre–COVID-19 (March 16, 2017, to March 15, 2020) and (2) COVID-19 (June 16, 2020, to March 15, 2023), were defined. We excluded treatment courses delivered in the first 3 months of COVID-19 because this was a transition period for the modified approach. Data were retrieved through treatment notes and medical record review. Modified anesthesia practice during COVID-19 included (1) preoxygenation with a Tavish mask, with BVM ventilation restricted to desaturation; (2) bed positioning at 30° to 45° inclination; and (3) decrease in succinylcholine and methohexital dosages. We restricted the assessment of complications to only the first acute course in the analyzed period and the first 9 treatments in each group to minimize potential bias due to higher exposure to complications in longer courses. Only individuals who received methohexital in the first treatment were included to minimize potential confounders associated with different adverse effect profiles for anesthetics. We also excluded individuals with missing weight measures or missing clinical outcomes. Missing data are reported in eTable 1 in Supplement 1. Treatments were delivered with a MECTA spECTrum 5000Q device (MECTA LLC), with starting electrode placement at the ECT psychiatrist’s discretion.

### Outcomes

The primary outcomes were the occurrence of individual clinical complications during the observation period and the rates of BVM ventilation in the COVID-19 group. We examined the following complications: (1) bradycardia or transient asystole, (2) insufficient initial sedation dosage, (3) residual muscle weakness, (4) postictal agitation (PIA), (5) prolonged seizure, and (6) aspiration, as evidenced by documentation in the psychiatrist or anesthesia section of the ECT procedure notes and the Clinical Global Impression (CGI)–Improvement scale.^22,23^ Each complication was coded as a binary variable if it occurred during a treatment course. BVM use was not assessed in the pre–COVID-19 group because this was a standard practice prior to COVID-19.

The secondary outcomes were rates of response and cognitive impairment. We assessed the CGI–Improvement item (CGI-I),^22^ with scores 4 to 7 grouped into the score of 4 (no change or worse). Treatment response was defined as a score of 1 to 2, whereas scores of 3 to 4 were considered nonresponse. For missing CGI-I scores (85 participants), the main author (C.W.E.) completed a record review for a proxy of CGI-I item scores, termed clinical note CGI (c-CGI), using previously published techniques.^24,25^ A detailed description of the c-CGI is reported elsewhere.^24^ A subset of 100 treatment courses with complete CGI-I scores were used to validate this approach; disagreements between the c-CGI and the CGI-I scores were reviewed by other authors (T.K. or J.P.) and their scores were used. When comparing response (scores 1-2) vs nonresponse (scores 3-4) in the CGI-I, the interrater agreement between CGI-I and c-CGI was good (Cohen κ = 0.73; quadratic weights).^26^ We measured cognitive impairment at the end of an acute course in a similar 4-point scale (1 = none, 2 = mild, 3 = moderate, and 4 = severe), with scores of 3 to 4 indicating significant cognitive impairment. Cognitive tolerability was poorly documented in medical records for 25% of reviewed scores. Interrater agreement was moderate for raw cognitive impairment scores (κ = 0.59; linear weights) and good for clustered scores (1-2 vs 3-4; κ = 0.77; quadratic weights).^26^

### Statistical Analyses

We reported the number and proportion of individuals who experienced each complication in both groups and BVM use in the COVID-19 group using descriptive methods. The Shapiro-Wilk test was used for normality testing of numerical variables, followed by Student *t* test or Mann-Whitney U Test for group comparison. For categorical variables, we used χ^2^ or Fisher exact test to compare the rates of complications, response rates, and cognitive impairment. We used odds ratios (ORs) and corresponding 95% CIs to further assess the association of COVID-19–related measures, especially reduced doses of anesthesia medications, with the outcomes of clinical complications, response, and cognitive impairment. For each main outcome, we created a multivariable logistic regression model with age, sex, primary diagnosis, admission status, baseline severity, and starting electrode placement as covariates. Given the small number of events for most primary outcomes, we removed the psychiatric diagnoses from the covariates because these variables are not likely to be associated with adverse outcomes. Baseline severity was determined by CGI-Severity^22^ scores, by grouping scores of 3 of 5 as moderate and scores of 6 to 7 as severe. Statistical tests were 2-tailed with an α of .05. The diagnoses of mixed episode and manic episode within bipolar disorder were collapsed into a single diagnostic category due to sparse data. The techniques used for internal model validation are provided in the eMethods in Supplement 1. The missing CGI score for baseline severity and missing CGI-I scores were completed by 1 author (C.W.E.) using the c-CGI (eTable 1 in Supplement 1). Following the primary outcome analysis, we conducted post hoc analyses as appropriate. Finally, we performed a second sensitivity analysis of primary outcomes including individuals initially excluded from the main analysis. Statistical analyses were performed with R software version 4.3.2 (R Project for Statistical Computing)^27^ from February to December 2024.

## Results

The study sample comprised 616 individuals (median [IQR] age, 45.0 [31.0-59.0] years; 342 female [55.5%]), of whom 362 were in the pre–COVID-19 group and 254 were in the COVID-19 group. Of the 740 individuals who started an acute course of ECT during the study period, 3 were excluded due to lack of reported outcomes, 78 were excluded because they received a general anesthetic other than methohexital, and 45 were excluded due to missing weights. Demographic and clinical characteristics of both groups were similar, except for a higher proportion of individuals with catatonia in the COVID-19 group than the pre–COVID-19 group (23 individuals [9.1%] vs 12 individuals [3.3%]; *P* = .002) (Table 1). The rates of early treatment dropouts (defined as having received ≤3 ECT treatments) were similar between groups (28 individuals in the pre–COVID-19 group [7.7%] vs 19 individuals in the COVID-19 group [7.5%]; *P* = .91). There were no ECT-related deaths in the study period. Groups were similar in the proportion of electrode placement at first treatment (right unilateral vs bilateral) and starting charge, yet a switch to bilateral electrode placement was more frequent in the pre–COVID-19 group than the COVID-19 group (67 individuals [26.4%] vs 30 individuals [16.8%]; *P* = .02). The COVID-19 group had a significantly lower median (IQR) starting dosage of methohexital (0.87 [0.78-1.00] mg/kg vs 0.82 [0.72-0.96] mg/kg; *P* = .007) and succinylcholine (0.53 [0.45-0.59] mg/kg vs 0.33 [0.27-0.39] mg/kg; *P* < .001) than the pre–COVID-19 group.

**Table 1.** Demographic and Clinical Characteristics and Treatment Variables of the Included Individuals

Variable	Participants, No. (%) (N = 616)		*P* value^a^
	Pre–COVID-19 group (n = 362)	COVID-19 group (n = 254)	
Demographics and clinical characteristics			
Age, median (IQR), y	44.0 (30.0-58.8)	46.0 (31.0-60.0)	.29^b^
Sex			
Female	203 (56.1)	139 (54.7)	.74^c^
Male	159 (43.9)	115 (45.3)	
CGI baseline severity			
Median (IQR)	5.0 (5.0-6.0)	5.5 (5.0-6.0)	.08^b^
Mean (SD)	5.34 (0.86)	5.47 (0.82)	
Baseline severe illness (CGI scores 6-7)	161 (44.5)	127 (50.0)	.18^c^
Inpatient status	259 (71.5)	171 (67.3)	.26^c^
Primary diagnosis			
Unipolar depression	184 (50.8)	124 (48.8)	.62^c^
Bipolar depression	43 (11.9)	35 (13.8)	.48^c^
Depression with psychosis	39 (10.8)	27 (10.6)	.95^c^
Schizophrenia	44 (12.2)	34 (13.4)	.65^c^
Catatonia	12 (3.3)	23 (9.1)	.002^c^
Schizoaffective depression	27 (7.5)	14 (5.5)	.34^c^
Schizoaffective mania	14 (3.9)	12 (4.7)	.60^c^
Bipolar mixed	4 (1.1)	4 (1.6)	.72^d^
Bipolar mania	3 (0.8)	3 (1.2)	.69^d^
Bipolar mixed/mania	7 (1.9)	7 (2.8)	.50^c^
Other	17 (4.7)	7 (2.8)	.22^c^
Treatment variables			
No. of treatments, median (IQR)	11.0 (7.0-15.0)	12.0 (8.0-16.0)	.10^b^
No. of early dropouts (≤3 treatments)	28 (7.7)	19 (7.5)	.91^c^
No. of RUL starts	254 (70.2)	179 (70.5)	.93^c^
Starting charge for RUL, median (IQR), mC	19.2 (19.2-38.4)	19.2 (19.2-38.4)	.99^b^
No. of BL starts	108 (29.8)	75 (29.5)	.93^c^
Starting charge for BL, median (IQR), mC	160.0 (96.0-240.0)	128.0 (96.0-192.0)	.06^b^
No. of RUL-BL electrode switches^e^	67 (26.4)	30 (16.8)	.02^c^
Starting methohexital dose, median (IQR), mg/kg	0.87 (0.78-1.00)	0.82 (0.72-0.96)	.007^b^
Starting succinylcholine dose, median (IQR), mg/kg	0.53 (0.45-0.59)	0.33 (0.27-0.39)	<.001^b^

Abbreviations: BL, bilateral; CGI, Clinical Global Impression; RUL, right unilateral.

^a^ Statistical significance for all tests was set at *P* < .05.

^b^ Wilcoxon rank sum test.

^c^ χ^2^ test.

^d^ Fisher exact test.

^e^ One individual in the COVID-19 group had an electrode switch to bifrontal placement. All others had a bitemporal electrode switch.

### Outcomes

There was no difference between the pre–COVID-19 and COVID-19 groups for most complications (Table 2). The COVID-19 group had a higher proportion of insufficient initial sedation dosage (15 individuals [4.1%] vs 21 individuals [8.3%]; *P* = .03) and PIA (33 individuals [9.1%] vs 55 individuals [21.7%]; *P* < .001) than the pre–COVID-19 group. Adjusted analyses further supported these findings with higher odds of insufficient initial sedation dosage (OR, 2.16; 95% CI, 1.09-4.38) and PIA (OR, 2.81; 95% CI, 1.76-4.56) in the COVID-19 group than the pre–COVID-19 group (Table 2). BVM was required for 35 individuals in the COVID-19 group (13.8%). In a post hoc analysis, we investigated anesthetic agents in subsequent treatments in the subset of individuals who experienced PIA and found that this complication occurred during methohexital use in all cases.

**Table 2.** Rates of Clinical Complications, Response, Cognitive Impairment, and Midazolam Use in the Included Individuals

Outcome	Participants, No. (%) (N = 616)		*P* value^a^	OR (95% CI)^b^
	Pre–COVID-19 (n = 362)	COVID-19 (n = 254)		
Primary outcomes: complications				
Bradycardia or transient asystole	17 (4.7)	14 (5.5)	.65^c^	1.20 (0.57-2.50)
Insufficient initial sedation dosage	15 (4.1)	21 (8.3)	.03^c^	2.16 (1.09-4.38)
Postictal agitation	33 (9.1)	55 (21.7)	<.001^c^	2.81 (1.76-4.56)
Prolonged seizure	36 (10.0)	27 (10.6)	.78^c^	1.24 (0.71-2.16)
Residual muscle weakness	24 (6.6)	18 (7.1)	.83^c^	1.06 (0.55-2.02)
Aspiration	2 (0.6)	0	.52^d^	NA^e^
Bag-valve mask ventilation (COVID-19 group only)	NA	35 (13.8)	NA	NA
Secondary outcomes				
Response	246 (68.0)	181 (71.3)	.38^c^	1.15 (0.80-1.65)^f^
Cognitive impairment	75 (20.7)	49 (19.3)	.66^c^	0.95 (0.63-1.43)^f^
Exploratory (post hoc)				
Headaches	46 (12.7)	36 (14.2)	.60^c^	1.27 (0.77-2.09)
Myalgias	44 (12.2)	41 (16.1)	.16^c^	1.43 (0.89-2.29)
Midazolam use	66 (18.2)	69 (27.2)	.008^c^	1.76 (1.18-2.62)

Abbreviations: NA, not applicable; OR, odds ratio.

^a^ Statistical significance for all tests was set at *P* < .05.

^b^ Adjusted for age, sex, electrode placement, inpatient status, and psychiatric illness severity. The reference group is the pre–COVID-19 group.

^c^ χ^2^ test.

^d^ Fisher exact test.

^e^ The regression model failed to converge due to a small number of observations.

^f^ Adjusted additionally for psychiatric diagnoses.

Given the differences in dosing of anesthetic agents between groups, we further explored the rates of myalgias and headaches, common adverse effects potentially associated with succinylcholine^28^ and methohexital,^29^ respectively. We failed to find a statistically significant difference between the pre–COVID-19 and COVID-19 groups in the rates of myalgias (44 individuals [12.2%] vs 41 individuals [16.1%]; *P* = .16) and headaches (46 individuals [12.7%] vs 36 individuals [14.2%]; *P* = .60) (Table 2). In a third post hoc analysis, we compared the proportion of midazolam use, a typical treatment of PIA,^21,30^ and found that this was higher in the COVID-19 group than the pre–COVID-19 group (66 individuals [18.2%] vs 69 individuals [27.2%]; *P* = .008) (Table 2). We additionally explored starting succinylcholine and methohexital doses within the subgroup of individuals with PIA and those without PIA across both groups. We found that individuals with PIA had a significantly lower succinylcholine dosage (median [IQR], 0.38 [0.31-0.51] mg/kg vs 0.45 [0.35-0.55] mg/kg; *P* < .001), with no difference for methohexital dosage (median [IQR], 0.84 [0.72-0.94] mg/kg vs 0.86 [0.75-0.99] mg/kg; *P* = .10). Finally, a sensitivity analysis including individuals excluded from the final cohort showed similar results for clinical complications and midazolam use (eAppendix 1, eTable 2, and eTable 3 in Supplement 1).

For secondary outcomes, response rates were similar in both groups (246 individuals [68.0%] in the pre–COVID-19 and 181 individuals [71.3%] in the COVID-19 group; *P* = .38) (Table 2). The groups did not differ in the rates of clinically relevant cognitive impairment (75 individuals [20.7%] in the pre–COVID-19 group vs 49 individuals [19.3%] in the COVID-19 group; *P* = .66). We found no association of COVID-19–related anesthesia modifications with the odds of response when controlling for potential confounders (OR, 1.15; 95% CI, 0.80-1.65) (Table 2). Similarly, we did not identify an association of COVID-19–related anesthesia modifications with the odds of experiencing clinically significant cognitive impairment after adjusting for potential confounders (OR, 0.95; 95% CI, 0.63-1.43) (Table 2). Sensitivity analyses including excluded individuals (737 individuals) supported these findings, with similar rates of response and cognitive impairment in both groups in both unadjusted and adjusted analyses (eAppendix 2, eAppendix 3, the eFigure, and eTables 3-5 in Supplement 1).

## Discussion

To our knowledge, this cohort study is the first to report results on the safety, cognitive tolerability, and clinical outcomes with ECT in a large cohort that received modified anesthesia practice during COVID-19. Our modified anesthesia practice allowed for a safe reduction in BVM use. Clinical complications were largely similar, except for a higher incidence of PIA and an increased rate of insufficient initial sedation dosage in the COVID-19 group. These findings were replicated in a sensitivity analysis including individuals receiving other anesthetics. Nevertheless, these findings were not associated with markers of poorer tolerability in the COVID-19 group because the ECT course duration, the proportion of early dropouts, and the prevalence of common adverse effects of headaches and myalgias were comparable between groups. Furthermore, modified anesthesia procedures were not associated with different clinical and cognitive outcomes. Taken together, our results provide important information on the tolerability and effectiveness of ECT following anesthetic dosage reduction and could help inform safe modifications to ECT practice in future pandemics with consideration to monitoring the emergence of PIA.

The anesthesia practice modifications during the COVID-19 pandemic reduced the need for BVM use from standard of care (ie, 100%) to only 13.8% of individuals in the COVID-19 cohort. This was an important measure to promote patient and staff safety and allowed the operation of the ECT service in a restricted environment. Preoxygenation with a nonrebreather mask may also have contributed to reduced BVM use because this was found to be comparable to BVM ventilation in 1 study.^31^ It is possible that reduced BVM ventilation could have had additional benefits including decreased risk of aspiration.^32,33^ Lower methohexital starting dosage in the COVID-19 group was likely associated with higher rates of insufficient initial sedation dosage compared with the pre–COVID-19 group. Adequate sedation was achieved through the administration of an additional methohexital bolus to ensure adequate sedation prior to treatment, followed by an increase in methohexital dosage in subsequent treatments. The median succinylcholine starting dosage (0.33 mg/kg) aligns with findings from a previous study,^16^ which reported a reduction in succinylcholine dosage to 0.3 to 0.4 mg/kg during COVID-19 without an increase in adverse effects and complications, with the advantage of shorter recovery times for some patients. However, the study^16^ did not provide a detailed comparison of rates of complications or treatment response.

PIA is a relatively common complication, with incidence estimated between 5% to 36% of patients receiving ECT.^30,34,35,36,37^ Our typical protocol for PIA management involves the administration of midazolam or additional methohexital bolus^30,37,38,39^ followed by midazolam prophylaxis after subsequent treatments because PIA has high rates of recurrence.^30,36,38,40^ The higher proportion of midazolam use in the COVID-19 group may be associated with the increased incidence of PIA in this group, although additional COVID-19–related factors may have contributed to the higher midazolam use. PIA has been associated with lower succinylcholine dosage,^34,41,42,43^ with a hypothesis that inadequate neuromuscular blockade leads to an increase in lactate levels and lactate-induced panic.^30,34,41^ Some studies have reported resolution of PIA after an increase in succinylcholine dose,^34,41^ with 1 study^41^ reporting higher lactate levels in participants with PIA that normalized after dose increase. Lower methohexital dosage has also been associated with emergence of PIA in some studies.^39,43^ Other studies, however, did not find an association of doses of general anesthetics and succinylcholine with the incidence of this complication^36,44^ and failed to identify factors associated with PIA.^36^ In our study, the role of lower methohexital dosage in the emergence of PIA was unclear because this was not different between individuals with PIA and those who did not experience this complication. Overall, it is possible that the lower succinylcholine dosage in the COVID-19 group was associated with the higher rates of PIA. Additionally, it is also possible that reduced BVM use could have led to hypercapnia with subsequent increased anxiety and PIA in some patients.^30,45^ Future research should explore the role of reduced anesthesia medications and alternative preoxygenation techniques in the emergence of PIA.

Importantly, reduced doses of succinylcholine and methohexital were not associated with a decrease in ECT effectiveness. Response rates of 68% to 71% were comparable between groups and consistent with previous ECT studies.^24,46,47,48,49,50,51,52^ Furthermore, lower succinylcholine and methohexital doses were not associated with differences in cognitive tolerability. Significant cognitive impairment was present in approximately one-fifth of individuals in each group and was comparable to prior studies.^50,53,54^ The similar clinical and cognitive findings are reassuring because our modified anesthesia practice allowed for continuation of ECT provision in our center while many services were shut down in Canada and worldwide.^1,2,11^ Future research should investigate the ideal anesthetic dosage and the threshold for BVM ventilation in ECT. Finally, another future direction could examine preoxygenation with a face mask as a substitute for BVM ventilation.

### Limitations

This study has several limitations. Given the retrospective design of this study, conclusions about causality cannot be definitively established. Although we conducted a comprehensive search to identify complications, some occurrences might not have been detected. Moreover, potential complications occurring later in the ECT course were not considered because only the first 9 treatments were included. Importantly, our COVID-19–related interventions were multifactorial, including modifications in preoxygenation with higher threshold for BVM use, inclined bed rest, and lower dosage of methohexital and succinylcholine. Therefore, associations of reduced anesthesia dosing cannot be isolated from other cointerventions. It is possible that differences in PIA rates may be more accurately attributed to interactions between interventions implemented as part of the COVID-19 response. Furthermore, cohort effects that were not directly measurable in our study could also play a role. For instance, there may have been a higher prevalence of recreational drug use in the COVID-19 group, given reports indicating an increase in substance use during the COVID-19 pandemic,^55^ and substance use might increase the risk of PIA.^56^ Additionally, the anesthesia-ECT time interval was not assessed in this study. Modifications to preoxygenation protocols during COVID-19 may have led to a shortened anesthesia-ECT time interval, which is a known factor associated with seizure quality and cognitive impairment,^57,58^ although the magnitude of this association is larger in patients receiving propofol anesthesia than in patients receiving anesthetics with less prominent anticonvulsant properties such as methohexital and etomidate. Furthermore, potential confounders, such as other medications and clinical comorbidities, were not considered and may influence our findings. Another limitation is the use of CGI-I for assessment of cognitive impairment because this might not be sensitive enough to detect smaller yet significant differences in cognitive outcomes between groups. This assessment may also be inaccurate due to poor documentation of cognitive outcomes for some individuals. Additionally, we consider our post hoc analyses exploratory, and their results should be interpreted with caution.

## Conclusions

In this retrospective cohort study of ECT outcomes following COVID-19–related modifications to anesthesia practice, reduced methohexital and succinylcholine dosages were associated with decreased need for BVM ventilation with comparable safety, tolerability, and clinical outcomes other than increases in PIA and insufficient initial sedation dosage. Given the observational retrospective design with a large, clinically representative sample, we anticipate our results may be generalizable to other ECT settings. In sum, this study offers guidance for safe anesthesia practice in ECT during future pandemics and warrants additional investigation into the association of low succinylcholine dosage with the emergence of PIA.

## Notes

Supplement 1. **eTable 1.** Number of Missing Observations for Each Variable for All Individuals Treated in Study Period (n=740) and the Imputation Methods Used **eMethods.** Description of the Techniques Used for Internal Validation of Logistic Regression Models **eAppendix 1.** Assessment of Clinical Complications in the Individuals Included in the Sensitivity Analysis (n=740) **eTable 2.** Demographic and Clinical Characteristics and Treatment Variables of Individuals Included in the Sensitivity Analysis (n=740) **eTable 3.** Rates of Clinical Complications, Response and Cognitive Impairment in Individuals Included in the Sensitivity Analysis (n=740)* **eAppendix 2.** Results of Multicollinearity Assessment, C-Statistic, Goodness-of-Fit Test of the Multivariable Logistic Regression of Response **eAppendix 3.** Results of Multicollinearity Assessment, C-Statistic and Goodness-of-Fit Test, for Multivariable Logistic Regression of Cognitive Impairment **eFigure.** Correlation Matrix of Coefficients Included in the Logistic Regression Model of Response **eTable 4.** Sensitivity Analysis: Logistic Regression of Response After Removal of Unipolar Depression Variable **eTable 5.** Sensitivity Analysis: Logistic Regression of Cognitive Impairment After Removal of Unipolar Depression Variable

Supplement 2.
Data Sharing Statement
