# Supplementary material for: Tolerability and Clinical Outcomes With Anesthesia Dose Reduction During Electroconvulsive Therapy
Source: JAMA Netw Open. 2025 Feb 27;8(2):e2462054. doi: 10.1001/jamanetworkopen.2024.62054 (PMC11868969; doi:10.1001/jamanetworkopen.2024.62054)
Supplement: Supplement 1. — eTable 1. Number of Missing Observations for Each Variable for All Individuals Treated in Study Period (n=740) and the Imputation Methods Used eMethods. Description of the Techniques Used for Internal Validation of Logistic Regression Models eAppendix 1. Assessment of Clinical Complications in the Individuals Included in the Sensitivity Analysis (n=740) eTable 2. Demographic and Clinical Characteristics and Treatment Variables of Individuals Included in the Sensitivity Analysis (n=740) eTable 3. Rates of Clinical Complications, Response and Cognitive Impairment in Individuals Included in the Sensitivity Analysis (n=740)* eAppendix 2. Results of Multicollinearity Assessment, C-Statistic, Goodness-of-Fit Test of the Multivariable Logistic Regression of Response eAppendix 3. Results of Multicollinearity Assessment, C-Statistic and Goodness-of-Fit Test, for Multivariable Logistic Regression of Cognitive Impairment eFigure. Correlation Matrix of Coefficients Included in the Logistic Regression Model of Response eTable 4. Sensitivity Analysis: Logistic Regression of Response After Removal of Unipolar Depression Variable eTable 5. Sensitivity Analysis: Logistic Regression of Cognitive Impairment After Removal of Unipolar Depression Variable [file jamanetwopen-e2462054-s001.pdf]

Supplemental Online Content

Espinola CW, Kaster TS, Prillo J, et al. Tolerability and clinical outcomes with anesthesia dose reduction during electroconvulsive therapy. *JAMA Netw. Open*. 2025;8(2):e2462054. doi:10.1001/jamanetworkopen.2024.62054

eTable 1. Number of Missing Observations for Each Variable for All Individuals Treated in Study Period (n=740) and the Imputation Methods Used

eMethods. Description of the Techniques Used for Internal Validation of Logistic Regression Models

eAppendix 1. Assessment of Clinical Complications in the Individuals Included in the Sensitivity Analysis (n=740)

eTable 2. Demographic and Clinical Characteristics and Treatment Variables of Individuals Included in the Sensitivity Analysis (n=740)

eTable 3. Rates of Clinical Complications, Response and Cognitive Impairment in Individuals Included in the Sensitivity Analysis (n=740)*

eAppendix 2. Results of Multicollinearity Assessment, C-Statistic, Goodness-of-Fit Test of the Multivariable Logistic Regression of Response

eAppendix 3. Results of Multicollinearity Assessment, C-Statistic and Goodness-of-Fit Test, for Multivariable Logistic Regression of Cognitive Impairment

eFigure. Correlation Matrix of Coefficients Included in the Logistic Regression Model of Response

eTable 4. Sensitivity Analysis: Logistic Regression of Response After Removal of Unipolar Depression Variable

eTable 5. Sensitivity Analysis: Logistic Regression of Cognitive Impairment After Removal of Unipolar Depression Variable

This supplemental material has been provided by the authors to give readers additional information about their work.

© 2025 Espinola CW et al. *JAMA Network Open*.

eTable 1. Number of missing observations for each variable for all individuals treated in study period (n=740)

Variable	Number of missing observations			Notes
	Pre-COVID group	COVID group	Total	
Weight	41	4	45	Individuals with missing weight measures were removed from the main analysis and included in the sensitivity analysis of primary outcomes
CGI-I scores	55	30	85	c-CGI was completed by CWE as a proxy for CGI-I for 82 patients. We removed 3 patients (all from the pre-COVID group) due to lack of clinical information to inform an assessment of response or cognition tolerability
Baseline CGI severity scores	1	0	1	Estimated by one author (CWE) using chart review

CGI, Clinical Global Impression; c-CGI: Clinical Note CGI; CGI-I: Clinical Global Impression-Improvement scale.

© 2025 Espinola CW et al. *JAMA Network Open*.

eMethods. Description of the techniques used for internal validation of logistic regression models

We calculated the goodness-of-fit for each model using Holsmer-Lemeshow test and assessed multicollinearity using variance inflation factor (VIF). In case of variables with VIF>5, we calculated a correlation matrix of coefficients and conducted sensitivity analysis of a second logistic regression model without these variables. For internal validation, we used the c-statistic, a measure of the area under the ROC curve, and further applied a bootstrap correction with 100 to correct for optimism

© 2025 Espinola CW et al. *JAMA Network Open*.

eAppendix 1. Assessment of clinical complications in the individuals included in the sensitivity analysis (n=740)

Similar to the main analysis, in a sensitivity analysis including additional individuals treated with other anesthetics and those with missing data (n=740; eTable 3), the COVID group continued to have a higher proportion of insufficient initial sedation dosage [19(4.7%) vs 30(9.0%), P=.02] and PIA [42(10.3%) vs 72(21.6%), P<.001], with no difference between groups for other complications (eTable 4). In this larger sample, the rates of myalgias and headaches were also similar between groups, whereas midazolam use was higher in the COVID group in both unadjusted and adjusted analyses (eTable 4).

© 2025 Espinola CW et al. *JAMA Network Open*.

eTable 2. Demographic and clinical characteristics and treatment variables of individuals included in the sensitivity analysis (n=740)

Variable	Pre-COVID group (n=407)	COVID group (n=333)	*P* Value[a]
Demographics and clinical			
Age	Median (IQR): 44.0 (31.0-59.0)<br>Mean (SD): 45.5 (17.1)	Median (IQR): 46.0 (31.0-61.0)<br>Mean (SD): 46.7 (17.0)	.12[b]
Sex	Females: 233 (57.3%)<br>Males: 174 (42.8%)	Females: 182 (54.7%)<br>Males: 151 (45.4%)	.48[c]
CGI Baseline severity	Median: 5.0<br>IQR: 5.0-6.0<br>Mean (SD): 5.28 (0.87)	Median: 5.0<br>IQR: 5.0-6.0<br>Mean (SD): 5.46 (0.80)	**.005[b]**
Severe illness at baseline (CGI scores 6-7)	167 (41.0%)	165 (49.6%)	**.02[c]**
Inpatient status	260 (63.9%)	227 (68.2%)	0.22[c]
Primary diagnosis (% within group)			
Unipolar depression	223 (54.8%)	158 (47.5%)	.05[c]
Bipolar depression	45 (11.1%)	45 (13.5%)	.31[c]
Depression with psychosis	40 (9.8%)	41 (12.3%)	.28[c]
Schizophrenia	44 (10.8%)	42 (12.6%)	.45[c]
Catatonia	12 (3.0%)	27 (8.1%)	**. 002**[c]
Schizoaffective, depressive episode	29 (7.1%)	23 (6.9%)	.91[c]
Schizoaffective, manic episode	14 (3.4%)	13 (3.9%)	.74[c]
Bipolar disorder, mixed episode	4 (1.0%)	4 (1.2%)	>.99[d]
Bipolar disorder, manic episode	3 (0.7%)	4 (1.2%)	.71[d]
Bipolar disorder, mania or mixed episode	7 (1.7%)	8 (2.4%)	.51[c]
Other	21 (5.2%)	10 (3.0%)	.15[c]
Treatment variables			
Number of treatments	Median: 11.0<br>IQR: 7.0-15.0	Median: 12.0<br>IQR: 8.0-15.0	.22[b]
Number of early dropouts (3 or less treatments)	35 (8.6%)	25 (7.5%)	.59[c]
Number of RUL starts	294 (72.2%)	233 (70.0%)	.50[c]
Starting charge for RUL	Median: 19.2 mC<br>IQR: 19.2-38.4 mC	Median: 19.2 mC<br>IQR: 19.2-38.4mC	.63[b]
Number of BL starts	113 (27.8%)	100 (30.0%)	.50[c]
Starting charge for RUL	Median: 144.0 mC<br>IQR: 96.0-240.0 mC	Median: 128.0 mC<br>IQR: 96.0-192.0 mC	.20[b]
Number of RUL-BL switches[e]	78 (19.2%)	40 (12.0%)	**.01[c]**
Starting anesthetic agent			
Methohexital	405 (99.5%)	257 (70.8%)	<.001[d]
Etomidate	0 (0%)	75 (2%)	<.001[d]
Propofol	2 (0.5%)	1 (0.3%)	>.99 [d]

BL, bilateral; CGI: Clinical Global Impression; RUL, right unilateral.
[a] Statistical significance for all tests were set at *P*<.05.
[b]Wilcoxon rank sum test.
[c]Chi-square test.
[d] Fisher's exact test.
[e] One individual in the COVID group was switched to bifrontal electrode placement. All others had a to bitemporal electrode switch.

© 2025 Espinola CW et al. *JAMA Network Open*.

eTable 3. Rates of clinical complications, response and cognitive impairment in individuals included in the sensitivity analysis (n=740)*

Outcome	Pre-COVID (n=407)	COVID (n=333)	*P* Value[a]	OR (95% CI)**
Primary – complications				
Bradycardia or transient asystole	17 (4.2%)	22 (6.6%)	.14[b]	1.67 (0.87-3.25)
Insufficient initial sedation dosage	19 (4.7%)	30 (9.0%)	**.02[b]**	2.16 (1.19-4.01)
Postictal agitation	42 (10.3%)	72 (21.6%)	**<.001**[b]	2.54 (1.68-3.90)
Prolonged seizure	39 (9.6%)	38 (11.4%)	.42[b]	1.33 (0.81-2.17)
Residual muscle weakness	26 (6.4%)	22 (6.6%)	.91[b]	1.06 (0.58-1.93)
BVM ventilation (COVID group only)	NA	41 (16.1%)	NA	NA
Aspiration	2 (0.5 %)	0 (0.0%)	.50[c]	NA****
Secondary				
Response	271 (67.1%)*	242 (72.7%)	.10[b]	1.22 (0.88-1.70)**
Cognitive Impairment	87 (21.5%)*	67 (20.1%)	.64[b]	0.97 (0.67-1.39)**
Exploratory (post hoc)				
Headaches	53 (13.0%)	49 (14.7%)	.51[b]	1.31 (0.84-2.05)
Myalgias	52 (12.8%)	52 (15.6%)	.27[b]	1.34 (0.87-2.05)
Midazolam use	75 (18.4%)	104 (31.2%)	**<.001**[b]	2.16 (1.52-3.09)

[a] Statistical significance for all tests was set at *P*<.05.
[b] Chi-square test.
[c] Fisher's exact test.
* Three individuals were excluded in the analyses of response and cognitive impairment in the pre-COVID group due to lack of reported outcomes.
** Adjusted for age, sex, electrode placement, inpatient status, and psychiatric illness severity. Reference group is pre-COVID.
*** Adjusted additionally for psychiatric diagnoses.
**** The regression model failed to converge due to a small number of observations.

© 2025 Espinola CW et al. *JAMA Network Open*.

eAppendix 2. Results of multicollinearity assessment, c-statistic, goodness-of-fit test of the multivariable logistic regression of response.

Assessment of multicollinearity in the logistic regression model of response yielded a VIF: 6.96 for unipolar depression. For this variable, the correlation matrix of coefficients indicated a weak positive correlation with female sex (0.22) and a weak-to-moderate negative correlation with other diagnoses (-0.15 to -0.37; eFigure 1). Starting RUL electrode had a moderate positive correlation with unipolar depression (0.39) and moderate negative correlation with schizophrenia (-0.40). Of note, there was not a significant association between unipolar depression variable and the odds of response (Wald test, *P*=.40). C-statistic was estimated at 0.626 and corrected to 0.653 after bootstrap procedure (95% CI: 0.612-0.690). Given half of individuals had a primary diagnosis of unipolar depression, we included this variable in our final model. Sensitivity analysis after removing unipolar depression showed again no association of COVID group and the odds of response (OR: 1.15, 95% CI: 0.80-1.66; *P*=.44; eTable 4), whereas inpatient status at baseline continued to be a significant predictor of response (OR: 1.72, 95% CI: 1.14-2.59; *P*=.01). Both models with and without unipolar depression fitted the data well (Hosmer-Lemeshow test, *P*=.55 and *P*=.68, respectively).

© 2025 Espinola CW et al. *JAMA Network Open*.

eAppendix 3. Results of multicollinearity assessment, c-statistic and goodness-of-fit test, for multivariable logistic regression of cognitive impairment

Similar to the response model, there was no significant association of unipolar depression and the odds of cognitive impairment (Wald test, *P*=.52). C-statistic was 0.585 with bootstrap corrected c-statistic estimated at 0.629 (95% CI: 0.577-0.678). Sensitivity analysis after removing unipolar depression (VIF: 6.19) yielded similar results, with no association between COVID group and the odds of cognitive impairment (OR: 0.95, 95% CI: 0.63-1.43; *P*=.80; eTable 5). None of the variables included in each model had a significant effect on the likelihood of experiencing cognitive impairment. Hosmer-Lemeshow test indicated a good fit for both models (*P*=.77 and *P*=.91, respectively).

© 2025 Espinola CW et al. *JAMA Network Open*.

eFigure. Correlation matrix of coefficients included in the logistic regression model of response

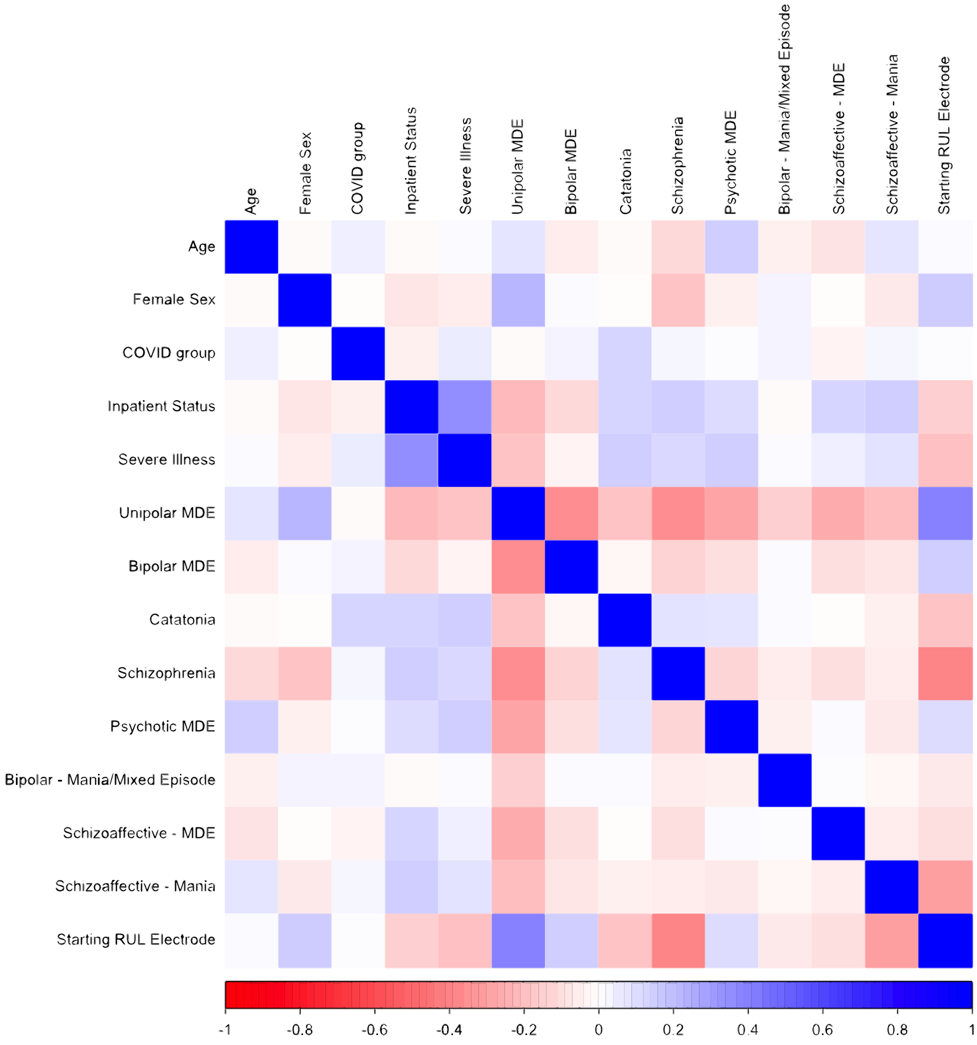

MDE, major depressive episode; RUL, right unilateral.

© 2025 Espinola CW et al. *JAMA Network Open*.

eTable 4. Sensitivity analysis: logistic regression of response after removal of unipolar depression variable

Variable	Odds Ratio	95% Confidence Interval	*P* Value[a]
Age	1.01	1.00-1.02	.44
Female sex	1.50	0.99-2.28	**.01**
COVID group	0.91	0.61-1.36	.44
Inpatient status	2.26	1.43-3.60	**.01**
Baseline severe illness (CGI score 6-7)	1.04	0.67-1.62	.85
Starting RUL electrode placement	1.19	0.69-2.03	.53
Bipolar depression	1.36	0.75-2.57	.32
Bipolar disorder, manic or mixed episode	2.09	0.52-13.94	.36
Depression with psychosis	1.27	0.63-2.71	.52
Catatonia	2.64	0.96-9.37	.09
Schizophrenia	1.04	0.52-2.14	.91
Schizoaffective, depressive episode	1.71	0.72-4.55	.25
Schizoaffective, manic episode	1.91	0.60-7.38	.30

CGI, Clinical Global Impression; RUL, right unilateral

[a] Statistical significance was set at $P<0.05$.

© 2025 Espinola CW et al. *JAMA Network Open*.

eTable 5. Sensitivity analysis: logistic regression of cognitive impairment after removal of unipolar depression variable

Variable	Odds Ratio	95% Confidence Interval	*P* Value[a]
Age	1.01	0.99-1.02	.32
Female sex	0.83	0.53-1.32	.44
COVID group	1.00	0.64-1.56	>.99
Inpatient status	0.74	0.44-1.25	.26
Baseline severe illness (CGI scores 6-7)	1.03	0.64-1.68	.89
Starting RUL electrode placement	0.60	0.34-1.08	.09
Bipolar depression	1.12	0.56-2.11	.74
Bipolar disorder, manic or mixed episode	0.79	0.12-3.20	.77
Depression with psychosis	1.41	0.68-2.83	.34
Catatonia	0.34	0.08-1.03	.09
Schizophrenia	0.75	0.33-1.64	.48
Schizoaffective disorder, depressive episode	0.45	0.13-1.25	.16
Schizoaffective disorder, manic episode	0.60	0.15-1.93	.42

CGI, Clinical global Impression; RUL, right unilateral
[a] Statistical significance was set at P<.05.

© 2025 Espinola CW et al. *JAMA Network Open*.
