# Supplementary material for: Tolerability and Clinical Outcomes With Anesthesia Dose Reduction During Electroconvulsive Therapy
Source: JAMA Netw Open. 2025 Feb 27;8(2):e2462054. doi: 10.1001/jamanetworkopen.2024.62054 (PMC11868969; doi:10.1001/jamanetworkopen.2024.62054)
Supplement: Supplement 2. — Data Sharing Statement [file jamanetwopen-e2462054-s002.pdf]

# Data Sharing Statement

Espinola. Tolerability and Clinical Outcomes With Anesthesia Dose Reduction During Electroconvulsive Therapy. *JAMA Netw Open*. Published February 27, 2025. doi:10.1001/jamanetworkopen.2024.62054

**Data**
**Data available:** Yes
**Data types:** Deidentified participant data
**How to access data:** Deidentified participant data along with data dictionaries will be made available, beginning 12 months and ending 3 years after publication of this article. Researchers can contact daniel.blumberger@camh.ca.
**When available:** With publication

**Supporting Documents**
**Document types:** None

**Additional Information**
**Who can access the data:** Researchers who provide a methodologically sound proposal that includes a protocol and a statistical analysis plan, and is not in conflict with the investigators' publication plan.
**Types of analyses:** A methodologically sound proposal approved by a research ethics board or institutional review board.
**Mechanisms of data availability:** To gain access, data requestors will need to sign a data access agreement.
